# Segmentation errors and intertest reliability in automated and manually traced hippocampal volumes

**DOI:** 10.1002/acn3.50885

**Published:** 2019-09-06

**Authors:** Benjamin H. Brinkmann, Hari Guragain, Daniel Kenney‐Jung, Jay Mandrekar, Robert E. Watson, Kirk M. Welker, Jeffrey W. Britton, Robert J. Witte

**Affiliations:** ^1^ Department of Neurology Mayo Clinic Rochester Minnesota; ^2^ Department of Physiology and Biomedical Engineering Mayo Clinic Rochester Minnesota; ^3^ Department of Neurology, Division of Child Neurology University of Minnesota Minneapolis Minnesota; ^4^ Division of Biomedical Statistics and Informatics Mayo Clinic Rochester Minnesota; ^5^ Department of Radiology Mayo Clinic Rochester Minnesota

## Abstract

**Objective:**

To rigorously compare automated atlas‐based and manual tracing hippocampal segmentation for accuracy, repeatability, and clinical acceptability given a relevant range of imaging abnormalities in clinical epilepsy.

**Methods:**

Forty‐nine patients with hippocampal asymmetry were identified from our institutional radiology database, including two patients with significant anatomic deformations. Manual hippocampal tracing was performed by experienced technologists on 3T MPRAGE images, measuring hippocampal volume up to the tectal plate, excluding the hippocampal tail. The same images were processed using NeuroQuant and FreeSurfer software. Ten subjects underwent repeated manual hippocampal tracings by two additional technologists blinded to previous results to evaluate consistency. Ten patients with two clinical MRI studies had volume measurements repeated using NeuroQuant and FreeSurfer.

**Results:**

FreeSurfer raw volumes were significantly lower than NeuroQuant (*P* < 0.001, right and left), and hippocampal asymmetry estimates were lower for both automatic methods than manual tracing (*P* < 0.0001). Differences remained significant after scaling volumes to age, gender, and scanner matched normative percentiles. Volume reproducibility was fair (0.4–0.59) for manual tracing, and excellent (>0.75) for both automated methods. Asymmetry index reproducibility was excellent (>0.75) for manual tracing and FreeSurfer segmentation and fair (0.4–0.59) for NeuroQuant segmentation. Both automatic segmentation methods failed on the two cases with anatomic deformations. Segmentation errors were visually identified in 25 NeuroQuant and 27 FreeSurfer segmentations, and nine (18%) NeuroQuant and six (12%) FreeSurfer errors were judged clinically significant.

**Interpretation:**

Automated hippocampal volumes are more reproducible than hand‐traced hippocampal volumes. However, these methods fail in some cases, and significant segmentation errors can occur.

## Introduction

Hippocampal atrophy is a recognized feature in temporal lobe epilepsy and a biomarker for mesial temporal sclerosis (MTS).^1^, ^2^, ^3^ Hippocampal volume loss is associated with neuronal loss and gliosis, which may be concentrated in specific subfields or broadly distributed.^4^ Resection of medial temporal structures in cases of MTS results in high rates of seizure freedom, and detection of hippocampal atrophy may help identify favorable candidates for epilepsy surgery.^5^, ^6^ Hippocampal volume loss may be detectable visually on qualitative MRI review; however, quantitative analysis may serve to verify and quantify the degree of hippocampal atrophy and asymmetry in temporal lobe epilepsy cases.^7^, ^8^

Detection of mild hippocampal atrophy requires accurate and reproducible quantitative measurement of hippocampal volumes as well as normative measures.^9^ Traditionally, hippocampal volume quantification has been performed by manual tracing of the hippocampal formations on individual MRI slices.^1^, ^2^ Recently, automated computer algorithms, capable of identifying the hippocampal boundaries based on anatomical atlases, have become available, and have shown promise toward objective volume measurements with minimal operator interaction.^10^, ^11^ NeuroQuant (CorTechs Labs, San Diego, California) is the first FDA‐approved (510[k]K061855) automated segmentation algorithm for clinical hippocampal volumetry and has gained widespread use in diagnosis and management of Alzheimer's disease and epilepsy. Automated hippocampal volume measurement (along with a large number of other brain structures) is also possible with FreeSurfer, an open‐source image analysis software package from the Martinos Center at Harvard University.^11^

We aimed to compare the accuracy of FreeSurfer and NeuroQuant automated segmentation measurement techniques with traditional manual hippocampal volumetry. Clinical assessment of hippocampal volumes requires accurate and reproducible measurements of left and right absolute volumes and asymmetry index. Asymmetry alone can be a sensitive indicator in cases of unilateral temporal lobe seizures, but detection of hippocampal atrophy in bitemporal epilepsy requires comparison to normative hippocampal volume measurements. Furthermore, quantification of the interstudy variation in hippocampal measurements is needed to establish the minimum detectable change using automated methods.

## Methods

**Figure 1:**
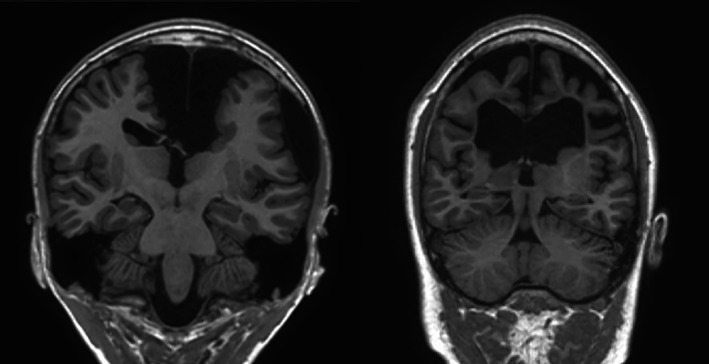
Gross anatomic deformations. Two patients, one with midline cysts (left) and another with ex vacuo ventricular dilation (right), were included in the study to assess the robustness of the automated algorithms to gross anatomic deformations distant from the hippocampus. Both automated segmentation algorithms failed on these two cases.

The Mayo Clinic Institutional Review Board reviewed and approved the use of retrospective MRI data for this study. Fifty seizure protocol MRI studies were identified retrospectively that had been acquired at our institution between 1 January 2015 and 31 January 2017, applying “Hippocampal Atrophy” or “Hippocampal Asymmetry” as search terms to our clinical radiology report database. Search results were reviewed to exclude “No Hippocampal Atrophy” or other phrases indicating entirely normal studies. Patients with previous resective surgery or gross cortical deformations were excluded, with the exception of two patients with such features included specifically to assess the robustness of the segmentation procedure: 1. Large interhemispheric cysts, 2. Global cerebral atrophy with ex vacuo ventricular dilation (Figure 1). All images were visually inspected, and images with excessive noise or motion artifacts were excluded. Images were excluded if no acceptable study was available. One of the MRI exams identified in our search was a follow‐up study of one patient already identified for the cohort; thus 49 patients in total were included in this study.

All MRI studies analyzed were acquired at 3 Tesla in a sagittal orientation with a T1‐weighted MPRAGE sequence provided by the vendor of NeuroQuant (TR = 6.5 msec TE = 2.5 msec, pixel dimensions 0.9375 × 0.9375 × 1.2 mm). Studies were acquired on both GE and Siemens systems. Manual hippocampus segmentations were performed by experienced 3D lab technologists using a dedicated hippocampal volume tracing program (Analyze MD, Biomedical Imaging Resource, Rochester MN). The manual segmentations are performed for left and right hippocampus separately in the coronal plane proceeding from the hippocampal head posteriorly to the tectal plate. Hence, the posterior tail of the hippocampus is omitted with this methodology, rendering volume lower than obtained by the two automated techniques. FreeSurfer version 5.3.0 was used to generate automated hippocampal segmentations, using default parameters. While FreeSurfer has the capability to take a T2‐weighted image as a command line input in addition to the T1‐weighted image to improve segmentation accuracy, this feature was not tested in this study. NeuroQuant version 2.0.1 was also used for algorithm‐generated hippocampal segmentations using the CorTech Labs processing receiver, as is done clinically at our institution. All three segmentation methods were applied to the same T1‐weighted images. No preprocessing was applied to the images prior to segmentation, but conversion from DICOM to nifti format was done for the FreeSurfer segmentations, as we occasionally had DICOM header errors when directly processing the DICOM files. NeuroQuant reports volumes in a normative percentile rank format along with raw volumes based on a large proprietary normative cohort of images. Normative percentiles are not produced by FreeSurfer directly but were computed using an open‐source calculator based on summary statistics from a large group of normal MRIs as a reference set (2790 subjects, 50.2% female, mean age 47.6 ± 21.8 year, range 18–94 year, 91% right handed, 53% acquired on 3T scanners).^12^

FreeSurfer and NeuroQuant algorithms produce color‐coded atlas overlays for validation of the hippocampal segmentations. The segmentation color images were reviewed by two radiologists (RJW, KW) to confirm the accuracy of hippocampal segmentations. Segmentation errors were graded from 1 (minor error) to 3 (major error). The clinical significance of segmentation errors was also assessed based on the severity of the error and the likelihood that the error could affect clinical decision‐making (e.g., volume overestimation could cause hippocampal sclerosis to be overlooked).

Ten patients from our cohort were selected at random to have manual segmentation repeated by two additional independent technologists in order to assess interobserver variation. No technologist segmented the same patient's images twice, and each technologist was blinded to the others' results. Manual segmentations were performed according to the normal clinical protocol at our institution, with segmentation proceeding to the tectal plate.

Ten patients in our cohort were identified with at least two T1‐weighted NeuroQuant MRI exams in our clinical records. These images had been acquired on different 3T MRI scanners (for most patients, GE PET‐MRI and Siemens Skyra scanners) as part of their clinical evaluation. NeuroQuant and FreeSurfer automated segmentation algorithms were applied to these repeated images, and the agreement of the hippocampal volumes was assessed.

Statistical calculations were performed using SAS version 9.4 (SAS Inc. Cary, NC) and Matlab (MathWorks, Natick MA). Intraclass correlation coefficients (ICCs) were calculated to assess the reproducibility of volume measurements within and among methods, in addition to conventional statistical tests. ICC values of reproducibility less than 0.4 are considered poor, 0.4 to 0.59 fair, 0.6 to 0.75 good, and ICC values above 0.75 excellent.

## Results

Forty‐nine patients (22 female) were identified after removal of one record from a patient with two MRI exams during the search period from our search results. Clinical and demographic features are summarized in Table 1. Mean age at MRI acquisition was 33.5 ± 20.2 years (range 0.75 to 72.5 years). Thirty‐nine patients in the cohort had a diagnosis of epilepsy, of which 30 were temporal lobe epilepsy (20 left‐sided, five right‐sided, five indeterminate or bitemporal), and the remainder generalized, extratemporal, or indeterminate localizations. Six patients had nonepileptic spells, and the remaining four patients had no relevant diagnosis. The subgroup of 10 patients (five female) whose MRIs underwent repeated manual hippocampal tracing had (mean ± SD.) age of 36.0 ± 15.6 years (range 19.0–69.5 years). Four patients in this group had left and two right temporal lobe epilepsy, one patient had nonlateralized temporal lobe epilepsy, one patient had nonlocalized epilepsy, one had nonepileptic spells, and one had no relevant diagnosis. The 10 additional patients with multiple MRI's had (mean ± SD) age 22.0 ± 7.1 years. Eight had temporal lobe epilepsy (three left‐sided, three right‐sided, and two nonlateralized), one had frontal lobe epilepsy, and one patient's epilepsy was not localized.

**Table 1:** Clinical and demographic summary of study cohort.

	Number	Age (year) Mean ± SD	Gender (% female)	Diagnosis of epilepsy	TLE ( Left, Right)
Volume comparison	49	33.5 ± 20.2	45%	39	30 (20, 5)
Repeated manual segmentation	10	36.0 ± 15.6	50%	9	7 (4, 2)
Repeated MRI	10	22.0 ± 7.1	50%	10	8 (3, 3)

**Figure 2:**
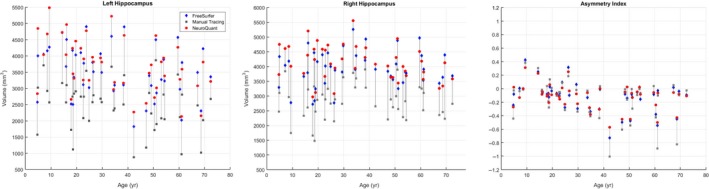
Hippocampal volume and asymmetry with age. Hippocampal volume measurements (*y*‐axis) in our cohort are plotted against subject age (*x*‐axis) for left and right hippocampus, and asymmetry indices (*y*‐axis) plotted against age (*x*‐axis). Manual tracing results are plotted as gray squares, FreeSurfer volumes are shown by blue diamonds, and NeuroQuant volumes are shown by red circles. Vertical gray lines connect FreeSurfer, NeuroQuant, and manual tracing measurements for individual subjects.

**Figure 3:**
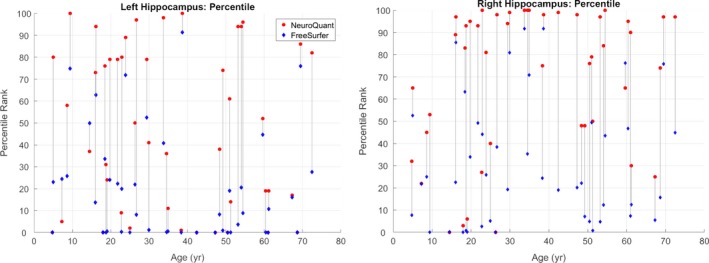
Hippocampal volume percentiles with age: hippocampal volume percentile ranks (*y*‐axis) in our cohort plotted against subject age (*x*‐axis) for left and right hippocampus. FreeSurfer hippocampal volumes are shown in blue diamonds, and NeuroQuant volumes are plotted as red circles. Vertical gray lines connect FreeSurfer and NeuroQuant volume percentile scores for individual subjects.

FreeSurfer failed on the two cases with gross deformations. NeuroQuant failed on four cases in total, the two cases with gross deformation, a 10‐month‐old male with mild left HC atrophy, and a 17‐month‐old male with mild right HC atrophy. Table 2 summarizes the left and right hippocampal volume measurements and calculated asymmetry index values for manual tracing, FreeSurfer, and NeuroQuant for the 45 patients in which all segmentation methods were completed successfully. FreeSurfer volumes for left and right hippocampus (LHC and RHC, respectively) were significantly lower than the volumes measured using NeuroQuant (*P* < 0.001, both measurements). FreeSurfer normalized percentile scores were also significantly lower than normalized percentiles for NeuroQuant (mean ± SD LHC: 48 ± 37% for NQ vs. 20 ± 25% for FS; RHC: 70 ± 33% for NQ vs. 30 ± 28% for FS, *P* << 0.0001 for both sides). Results remained significant when testing was repeated excluding the 11 pediatric subjects in the cohort for both LHC (mean ± SD percentile scores: 48 ± 37% for NQ vs. 17 ± 24% for FS) and RHC (76 ± 29% for NQ vs. 32 ± 28% for FS) with *P* << 0.0001. Raw volumes and asymmetry index values are plotted against subject age in Figure 2, while volume percentiles are plotted against subject age in Figure 3.

**Table 2:** Volumes and asymmetry indices–agreement between methods.

		Manual tracing^a^	FreeSurfer	NeuroQuant
Right HC (mm^3^)	Mean	2813	3926	4128
	St. Dev	584	603	585
Left HC (mm^3^)	Mean	2470	3526	3711
	SD	742	789	819
Asymmetry index	Mean	−0.1561	−0.1206	−01202
	SD	0.289	0.215	0.184

a Manual tracing excludes the hippocampal tail, resulting in systematically lower volumes compared to autosegmented measurements.

Because manual tracing segmentation did not cover the entire hippocampus, these volumes were a priori assumed to be lower (mean 32.7% LHC and 30.4% RHC differences were observed in our data) and were not tested for significance. Friedman's nonparametric test for repeated measures showed significant differences (*P* < 0.0001) in AsymIdx values between the three methods, and Wilcoxon rank sum tests showed significant differences between manual tracing and each automated method (*P* < 0.0001), but no difference between NeuroQuant, and FreeSurfer (Table 2).

The 10 patient group that underwent multiple MRI's were acquired with a mean ±SD inter‐scan interval of 120 ± 188 days (range 7–605 days). ICCs for repeated measurements are summarized in Table 3. Measurement reproducibility for left and right volume measurements was fair (0.4–0.59) for manual tracing, and excellent (>0.75) for FreeSurfer and NeuroQuant segmentation. Asymmetry index value reproducibility was excellent (>0.75) for manual tracing and FreeSurfer segmentation, and was fair (0.4–0.59) for NeuroQuant segmentation. The standard deviations for each patient's hippocampal volumes and AsymIdx values for each method were normalized to the mean values and averaged across the cohort, and are reported in Table 4.

**Table 3:** Intraclass correlation coefficients (ICCs) and confidence intervals for repeated volume measurements.

Method	Variable	ICC	Lower 95% CI	Upper 95% CI
Manual tracing	Left	0.5638	0.08956	0.8675
	Right	0.5783	0.08623	0.8748
	AI	0.9821	0.9617	0.9947
Freesurfer segmentation	Left	0.9368	0.8444	0.9819
	Right	0.8523	0.6663	0.9567
	AI	0.7584	0.4383	0.9302
NeuroQuant segmentation	Left	0.9346	0.8488	0.9809
	Right	0.9650	0.9199	0.9898
	AI	0.4985	0.0636	0.8328

Manual tracing segmentation was repeated by three different technologists on the same 10 patient images. NeuroQuant and FreeSurfer segmentation algorithms were run on repeated MRI scans for 10 patients. ICC values: <0.4 poor reproducibility, 0.4–0.59 fair, 0.6–0.75 good, <0.75 excellent reproducibility.

**Table 4:** Measurement variation–standard deviation as a percentage of mean.

	Left	Right	AI
FreeSurfer	2.38%	3.67%	3.97%
NeuroQuant	3.10%	2.22%	2.82%
Manual tracing	16.53%	15.06%	8.58%

**Figure 4:**
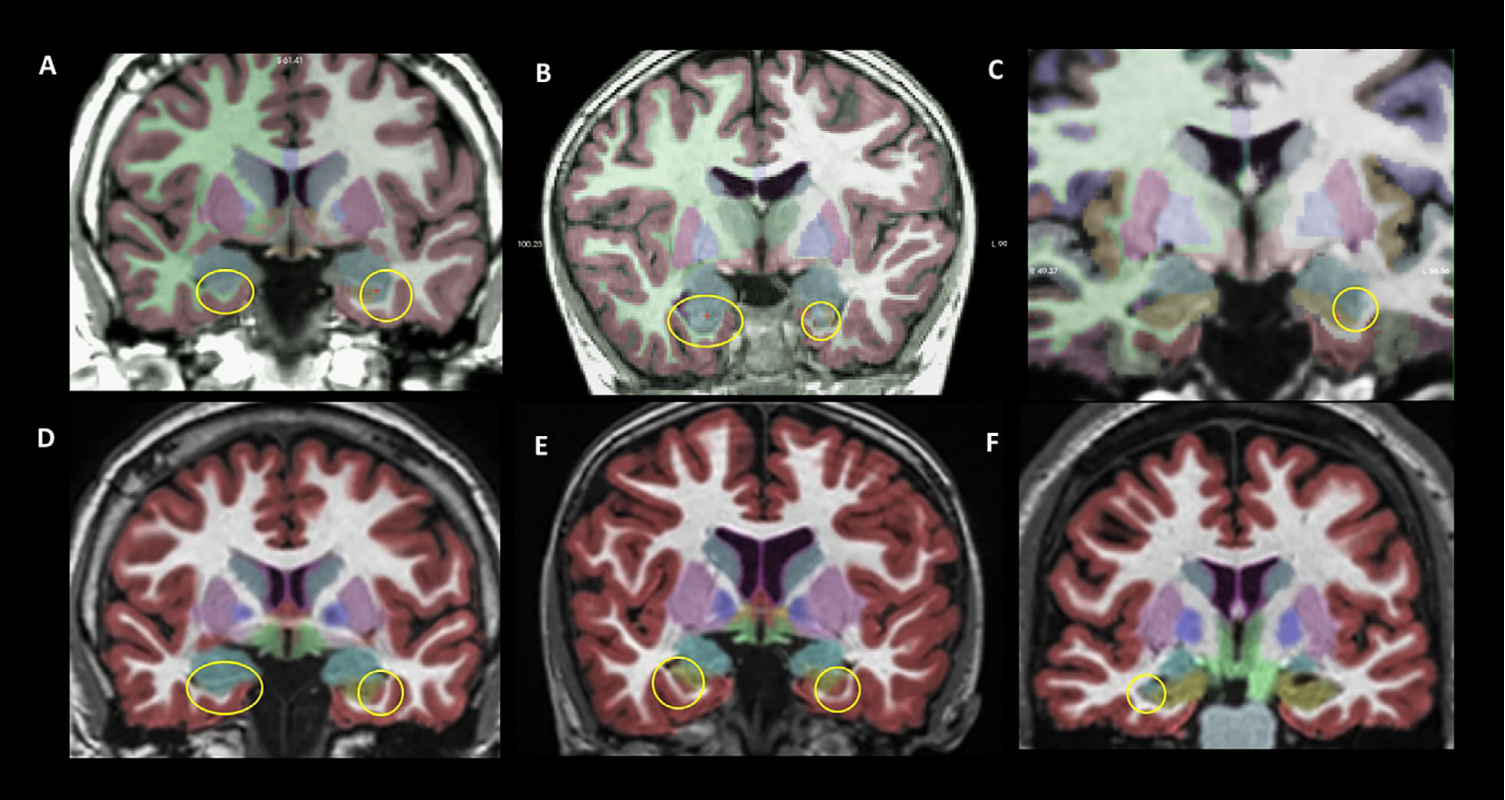
Segmentation errors–Examples of segmentation errors by FreeSurfer (top row) and NeuroQuant (bottom row), circled for emphasis. Transparent color overlays represent segmentation labels. Note that that structure colors differ between FreeSurfer and NeuroQuant. (A) FreeSurfer erroneously labeled the anterior right hippocampus as amygdala (blue), and labeled the lateral left anterior hippocampus as amygdala (B) FreeSurfer again labeled the right anterior hippocampus as amygdala (blue), and labeled the left mesial part of the anterior hippocampus as amygdala. (C) Here, FreeSurfer correctly segmented the right hippocampus (tan), but on the left side labeled a portion of the lateral hippocampus as amygdala. The FreeSurfer segmentation errors in (A–C) represent undersegmentation of the hippocampus and would produce artificially low hippocampal volumes. (D) NeuroQuant incorrectly labels the right anterior hippocampus as amygdala (blue), and labels the lateral anterior left hippocampus as cortex (red). (E) Here, NeuroQuant labels both the left and right lateral portions of the hippocampus (tan) as cortex (red). (F) NeuroQuant correctly identifies the left hippocampus (tan), amygdala (blue), and temporal neocortex (red) but erroneously labels the right lateral portion of the anterior hippocampus as amygdala (blue). The NeuroQuant errors in (D–F) represent undersegmentation of the hippocampus and would also produce artificially low hippocampal volumes.

Segmentation errors are summarized in Table 5, with examples of each type shown in Figure 4. FreeSurfer segmentation resulted in no errors rated as severe (category 3), while NeuroQuant resulted in three severe errors. In addition, FreeSurfer showed six clinically significant segmentation errors while NeuroQuant showed nine. These differences were not statistically significant (Fisher's exact test).

**Table 5:** Processing errors (*N* = 49).

	FreeSurfer	NeuroQuant
Run failures	2 (4%)	4 (8%)
Segmentation errors	27 (55%)	25 (51%)
Clinically significant errors	6 (12%)	9 (18%)
Clinically acceptable	41 (84%)	36 (73%)

## Discussion

This study showed significant differences between FreeSurfer and NeuroQuant hippocampal absolute volume measurements, and only moderate reproducibility in asymmetry index measurement for NeuroQuant. There was also significant variability in manually segmented cases among three experienced technologists with respect to absolute volume measures. Despite the poor reproducibility in manually traced volumes regarding absolute volume measures, asymmetry index showed excellent reproducibility, suggesting individual technologists were consistent in their over‐ or underestimation of the true hippocampal volumes. Overall, the high degree of variation in volume measurements (>15%) in hand‐measured images is concerning when the implications for selection of patients for surgery are considered. In contrast, both FreeSurfer and NeuroQuant segmentation produced low variation in volume measurements (<3.7%) despite the potential additional variations between images acquired on different MRI scanners at different times. Clearly, the measured variation in this study is a limited estimate of anticipated variation given the small number of scans for each patient. However, serial imaging in patients such as this is not widely available, and the difference observed between manual and automated methods in our study is large enough to provide confidence in this result.

The finding of significantly different hippocampal raw volume measurements between FreeSurfer and NeuroQuant on identical input images indicates that algorithm‐specific normative range measurements are necessary for accurate interpretation. While the magnitude of this difference was on average around 200 mm^3^ for both left and right sides, the difference was very consistent, and 32 of the 45 patients had larger volume measurements bilaterally with NeuroQuant. The highly significant differences in normalized volume percentile scores are surprising, however, as normalization to normal control ranges would be expected to compensate for consistent methodological differences between the segmentation techniques. This could be explained in part by the differences between the FreeSurfer and NeuroQuant normal control groups, although the normal control groups are large enough that we would expect these scales to represent population averages adequately. Figure 3 shows a surprising number of NeuroQuant values at the 100th and 0th percentiles, particularly for right hippocampus, and FreeSurfer percentile values more broadly distributed. The age range in the normal control groups may contribute to these differences: NeuroQuant's normal control range covers ages 3 to 100, while the FreeSurfer normal control image set covers ages 18 to 94. Our study cohort contained two subjects below age 3, and 11 subjects below age 18. However, with the 11 pediatric patients excluded from analysis, percentile score differences between NeuroQuant and FreeSurfer remained highly significant, suggesting there must be other differences in images or methodology to explain this.

In our analysis, AsymIdx values showed fair reproducibility with NeuroQuant while FreeSurfer and manual tracing showed excellent reproducibility. The greater number of segmentation errors with NeuroQuant may have contributed to this finding, and it should be noted as well that the 95% confidence intervals for these measurements overlap greatly. The large number of segmentation errors observed using atlas‐based segmentation algorithms, particularly on images with anatomic deformation, suggests that careful review and verification of segmented images are important steps in clinical practice. Multi‐atlas segmentation methods are developing rapidly^13^, ^14^ and hopefully will provide greater accuracy and robustness, particularly in the presence of natural or postsurgical deformations.

NeuroQuant has previously been benchmarked against neuroradiologist visual ratings for hippocampal atrophy and was found to have slightly better sensitivity and specificity.^15^ Prior studies comparing automated segmentation methods for hippocampal volumetry have found FreeSurfer to be more accurate than the open‐source FSL/FIRST algorithm^10^ when compared to manual tracing,^16^, ^17^ and found NeuroQuant raw volume measurements to be greater than FreeSurfer volumes by a margin similar to that observed in the present study.^18^ Multiple studies have benchmarked custom approaches to hippocampal segmentation^19^, ^20^, ^21^, ^22^ which show promise, but are not FDA approved or widely available to medical centers. No study to our knowledge has carefully assessed the rates and clinical acceptability of segmentation errors using these automated algorithms in the context of clinical decision‐making in epilepsy.

This study shows greater reliability in absolute volume measurements with NeuroQuant and FreeSurfer atlas‐based segmentation algorithms than manual tracing segmentation. These data support the use of such algorithms in assessing unilateral and bilateral hippocampal atrophy in epilepsy. This study also highlights a role for manual tracing segmentation in patients with large anatomic deformations, and the importance of careful review of algorithm segmentations to screen for errors before relying on volume measurement outputs for clinical reporting, given the clinically significant errors identified in 12% of the FreeSurfer and 18% of the NeuroQuant volumes. This study confirms systematic differences between FreeSurfer and NeuroQuant segmentation, and illustrates that normal control volume measurements generated by one approach should not be applied to measurements rendered by different volumetric techniques.

## Conflict of Interest

Guragain reports Grant support from Mr. and Mrs. David Hawk. The authors report no other conflict of interest directly relevant to this manuscript.
